# Metal-phenolic nanozyme as a ferroptosis inhibitor for alleviating cisplatin-induced acute kidney injury

**DOI:** 10.3389/fphar.2025.1535969

**Published:** 2025-04-01

**Authors:** Yunfeng Xiong, Huimin Kang, Yanping Rao, Xiayu Huang, Yang Zhu, Lixin Wei

**Affiliations:** ^1^ Department of Nephrology, Fujian Medical University Union Hospital, Fuzhou, China; ^2^ Department of Pediatrics, Fujian Medical University Union Hospital, Fuzhou, China; ^3^ CAS Key Laboratory of Soft Matter Chemistry, Department of Chemistry, University of Science and Technology of China, Hefei, China; ^4^ Fujian Institute of Clinical Immunology, Fuzhou, China

**Keywords:** nanozyme, acute kidney injury, ferroptosis, cisplatin, reactive oxygen species

## Abstract

**Introduction:**

Cisplatin-induced acute kidney injury (AKI) is primarily caused by oxidative stress from reactive oxygen species (ROS) accumulation. Developing ROS scavengers presents promising opportunities for preventing and treating this condition by targeting oxidative stress mechanisms.

**Methods:**

This study involves the fabrication of a metal-polyphenol self-assembled nanozyme (Fe@Ba) designed to inhibit ferroptosis through synergistic catalytic actions and antioxidant properties. The nanozyme is constructed using metal-polyphenol coordination-driven nanoprecipitation techniques. Its performance is evaluated *in vitro* using MTEC cells and *in vivo* within an AKI model, with assessments of catalytic activities, ROS depletion efficacy, antioxidant effects, and anti-ferroptotic mechanisms.

**Results:**

The Fe@Ba nanozyme demonstrates significant catalase (CAT) and superoxide dismutase (SOD)-like activities upon internalization by MTEC cells, effectively reducing high ROS levels in the AKI model. Baicalein (Ba), a traditional Chinese medicine component in the nanozyme, exhibits strong antioxidant properties, inhibits lipid peroxidation (LPO), upregulates reductive glutathione (GSH), and promotes glutathione peroxidase 4 (GPX4) expression, thereby inhibiting ferroptosis. Fluorescence imaging confirms effective renal accumulation of Cy5.5-labeled Fe@Ba nanozyme. *In vivo* experiments show the nanozyme reduces inflammation and significantly enhances survival rates in AKI models.

**Discussion:**

This study validates the concept of self-assembling nanozymes for AKI treatment and offers new insights into nanomedicine applications. The Fe@Ba nanozyme's ability to counteract inflammation-related damage and inhibit ferroptosis through multiple mechanisms highlights its therapeutic potential. The successful integration of traditional Chinese medicine components with nanotechnology represents an innovative approach to addressing cisplatin-induced AKI, suggesting broader applications for metal-polyphenol nanozymes in oxidative stress-related kidney diseases.

## Introduction

Acute kidney injury (AKI) is a critical global health issue that rapidly impairs kidney function, often resulting in tubular cell death and inflammation (Li et al., 2023; Jiang et al., 2024; Liu et al., 2020). It is frequently associated with severe illnesses and can be intensified by conditions such as reduced blood flow, sepsis, low blood pressure, and the overuse of certain antibiotics and chemotherapy drugs, including cisplatin (DDP) (Tang et al., 2023; Li et al., 2021a; Zhang et al., 2021a; Li et al., 2021b). DDP-induced AKI is a major clinical complication, and its development is closely tied to the buildup of reactive oxygen species (ROS) (Li et al., 2021a; Dai et al., 2020), which overwhelms the body’s antioxidant defenses (Zhao et al., 2021; Meng et al., 2018; Zhang et al., 2021b). These ROS can damage essential cellular components like lipids (Zhu et al., 2024a; Liu et al., 2015), nucleic acids (Zhu et al., 2023a; Liu et al., 2021), and proteins (Pan et al., 2024; Huang et al., 2024), leading to kidney dysfunction. While treatments like N-acetylcysteine are employed to counteract ROS and mitigate cisplatin-induced AKI, they are swiftly cleared by the immune system (Magner et al., 2022). In addition, antioxidant enzymes such as catalase (CAT) and superoxide dismutase (SOD) are promising candidates for clinical treatment of ROS-induced diseases (Chen et al., 2023; Scholz et al., 2021). Consequently, exploring artificial enzyme systems to address the challenges of scavenging ROS generation, and inhibiting ferroptosis, ultimately preventing cisplatin-induced kidney damage remains a substantial challenge.

Nanozymes, nanomaterials that mimic the functions of enzymes, are gaining recognition as a viable substitute for natural enzymes (Zhang et al., 2022; Zhu et al., 2023b; Jiang et al., 2019; Huang et al., 2019). Their appeal stems from their affordability, customizable catalytic properties, and enhanced stability (Chen and Arnold, 2020; Peng et al., 2024; Zhu et al., 2022; Zhu et al., 2021). Nanozymes have emerged as promising alternatives to natural enzymes, effectively bridging the unique intersection of nanotechnology and biomedicine (Zhu et al., 2023c; Xu et al., 2024; Zhu et al., 2024b). Metal-polyphenol nanozymes have been widely exploited in recent years (Liang et al., 2024a; Liang et al., 2024b). Previous studies have reported that various nanozymes with CAT- and SOD-like activities have been proven effective in treating AKI by neutralizing harmful ROS, which aids in the recovery of kidney function (Wang et al., 2022; Zhang et al., 2021c). While the potential of nanozymes in treating cisplatin-induced AKI is encouraging, several challenges remain (Li et al., 2024). Firstly, their relatively low catalytic efficiency hinders their effectiveness in treating AKI. Secondly, the ongoing process of ROS scavenging by nanozymes is not as effective as needed. Consequently, it is crucial to develop new nanozymes that are highly catalytically efficient and can continuously eliminate ROS, to address the issue of AKI in cancer patients treated with cisplatin.

Herein, we have developed a metal-polyphenol nanozyme, Fe@Ba, which is formed by the interaction of ferric ions (Fe^3+^) and the antioxidant compound baicalein (Ba) from traditional Chinese medicine. The polyphenol contains phenolic hydroxyl groups, which have more lone pair electrons, while the trivalent iron ion has vacant orbitals. Therefore, the phenolic hydroxyl groups can effectively coordinate with the iron ions to form nanoparticles. Fe@Ba inhibits lipid peroxidation (LPO), a process that contributes to cell death known as ferroptosis, by neutralizing harmful ROS and activating the antioxidant properties of baicalein. Fe@Ba nanozyme mimics the functions of natural enzymes, CAT and SOD, to convert toxic superoxide anions (·O_2_^−^) into harmless oxygen, thereby reducing inflammation and preventing ferroptosis. It also increases the expression of glutathione peroxidase 4 (GPX4), a key enzyme in the antioxidant defense system, further inhibiting ferroptosis. Fluorescence imaging reveals that the cyanine 5.5 (Cy5.5)-labeled Fe@Ba nanozyme effectively accumulates in the kidneys. Our *in vivo* experiments have shown that Fe@Ba can alleviate inflammation and improve survival rates in an AKI model, demonstrating its therapeutic potential. This research provides a proof-of-concept for the development of self-assembling nanozymes and offers new insights into the use of nanomedicine for treating AKI, highlighting their potential to mitigate the negative effects of inflammation.

## Results and discussions

**SCHEME 1:**
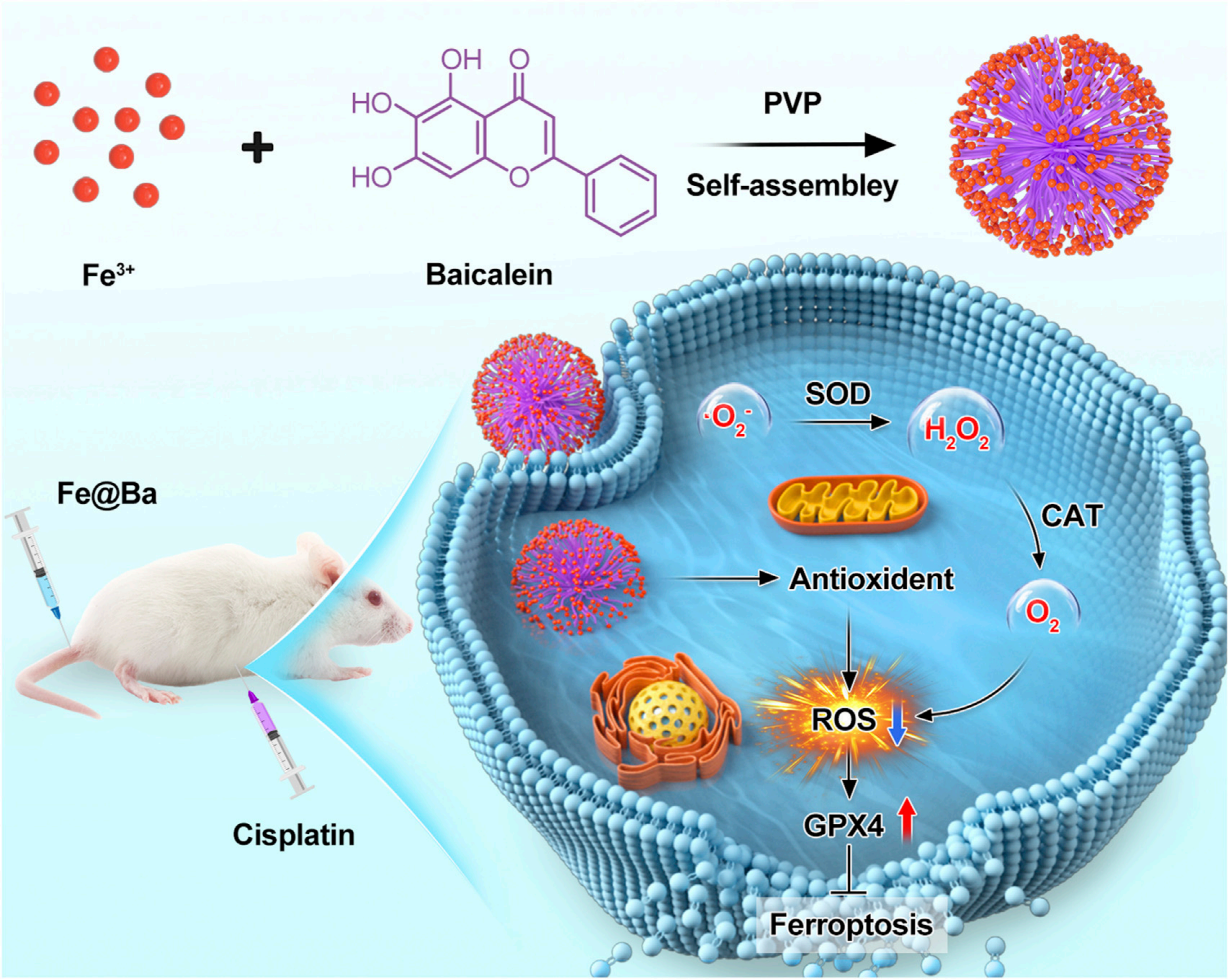
Schematic representation of the application of Fe@Ba nanozyme to inhibit ferroptosis in a cisplatin-induced AKI model.

**FIGURE 1:**
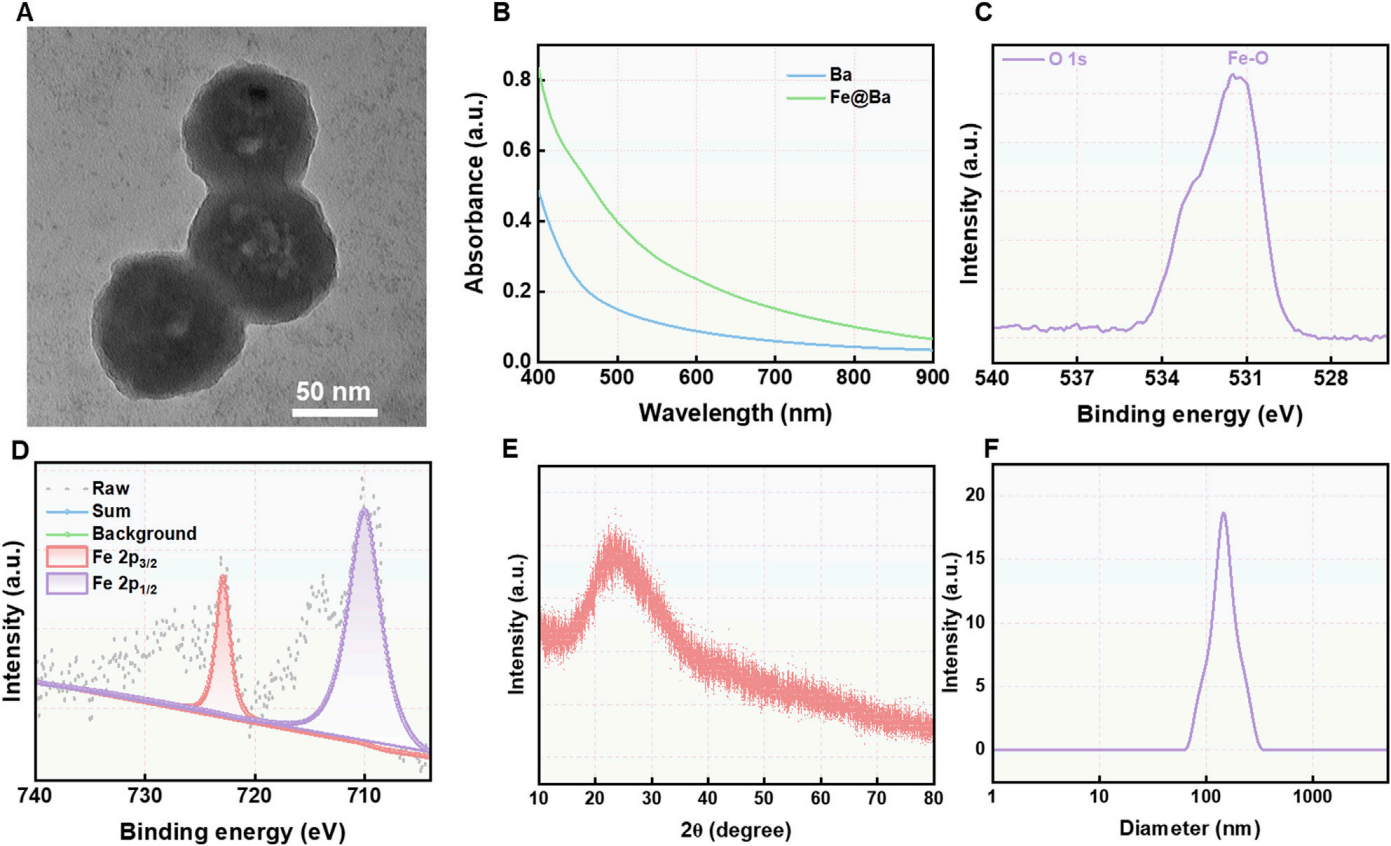
**(A)** TEM image of Fe@Ba nanozyme. **(B)** The DLS results of Fe@Ba nanozyme. **(C)** XPS spectrum of O 1s in Fe@Ba nanozyme. **(D)** High-resolution XPS spectrum of Fe 2p. **(E)** XRD spectrum of Fe@Ba nanozyme. **(F)** The UV-vis spectra of BDP and Fe@Ba nanozyme.

The synthesis of the Fe@Ba nanozyme was outlined in Scheme 1. We utilized a self-assembly method to combine Fe^3+^ ions with a metal-organic framework Ba in the presence of polyvinylpyrrolidone (PVP). As depicted in Figure 1A, the resulting Fe@Ba nanozyme, which was uniformly dispersed and approximately 50 nm in size, was directly visualized using transmission electron microscopy (TEM). The hydrodynamic size of the Fe@Ba nanozyme was measured with dynamic light scattering (DLS), as shown in Figure 1B. The zeta potential of Fe@Ba was −16.7 mV.

The high-angle annular dark-field scanning transmission electron microscopy (HAADF-STEM) images was employed to observed the element analysis (Supplementary Figure S1). Further analysis was conducted using X-ray photoelectron spectroscopy (XPS) to determine the oxidation states of iron and oxygen in the Fe@Ba nanozyme, with results presented in Figures 1D; Supplementary Figures S2, S3. The O 1s XPS spectrum revealed a peak at a binding energy of 531.4 eV, indicative of the presence of Fe-O bonds, as shown in Figure 1C. The high-resolution Fe 2p XPS spectrum split the Fe 2p peak into two components, corresponding to Fe^3+^ at 722.9 eV and Fe^2+^ at 710.1 eV, which are crucial for the nanozyme’s catalytic activity. The X-ray powder diffraction (XRD) spectrum indicated no distinct crystal pattern, suggesting that the iron in the Fe@Ba nanozyme had poor crystallinity, as shown in Figure 1E. Raman spectroscopy was employed to assess the degree of crystallinity, with results in Supplementary Figure S4. Ultraviolet-visible (UV-vis) spectroscopy confirmed the successful assembly of Ba and Fe^3+^, as illustrated in Figure 1F. Collectively, these findings confirmed the successful fabrication of the Fe@Ba nanozyme.

**FIGURE 2:**
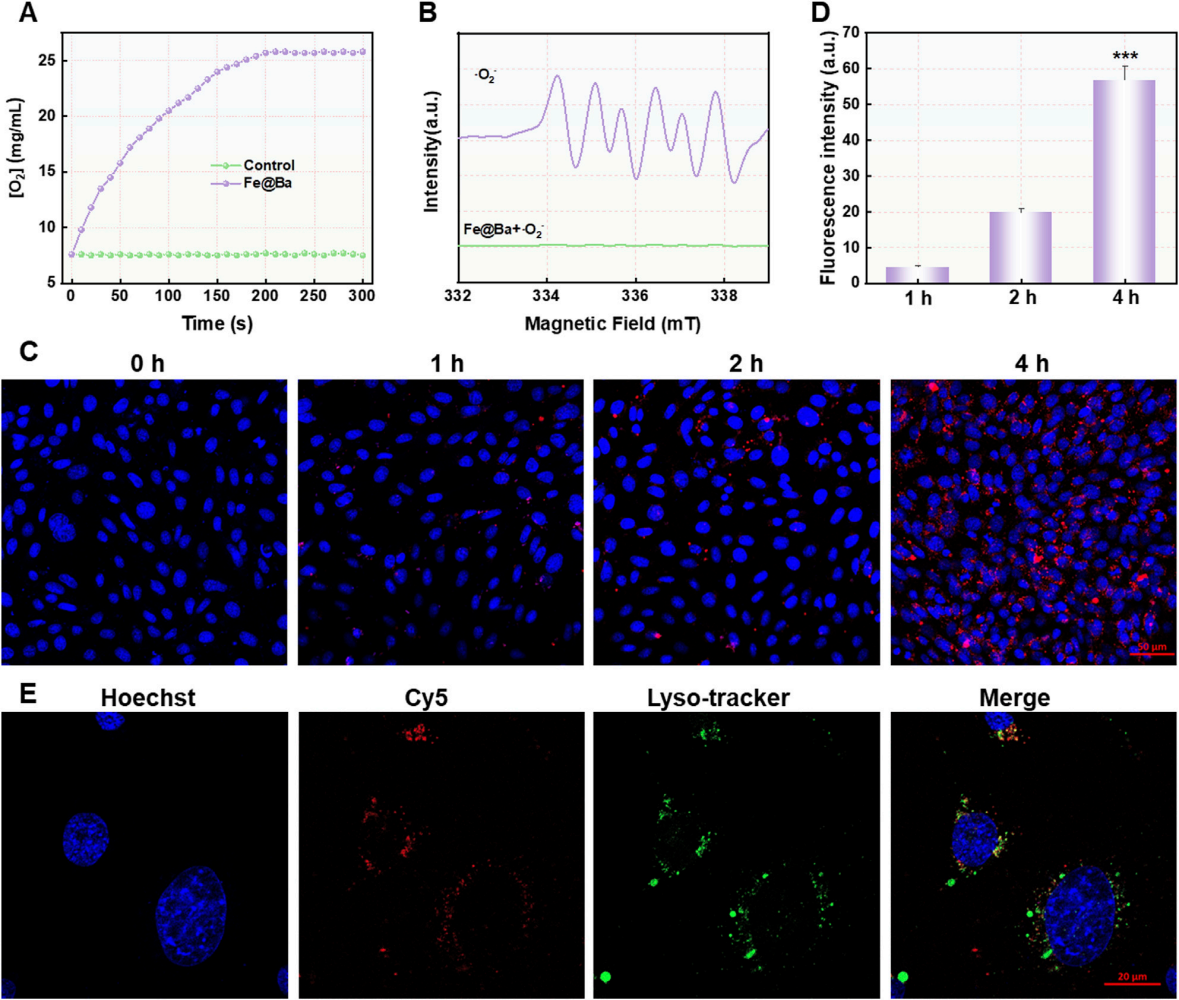
**(A)** The oxygen generation rate of H_2_O_2_ catalyzed by Fe@Ba nanozyme was assessed by dissolved oxygen analyzer. **(B)** ESR spectra of Fe@Ba nanozyme with DMPO as the spin trapper. **(C)** The CLSM images and **(D)** corresponding quantification of MTEC cells treated with Cy5.5-labeled Fe@Ba nanozyme. **(E)** The CLSM images revealed the colocalization of Cy5.5-labeled Fe@Ba nanozyme with the lysosomes of MTEC cells.

Motivated by the potential of multivalent iron elements, we investigated the catalytic capabilities of the Fe@Ba nanozyme. We assessed its catalase-like activity by measuring its ability to convert the highly toxic H_2_O_2_ into the non-toxic O_2_, which helps to reduce oxidative stress caused by ROS, as shown in Figure 2A. Furthermore, we determined SOD-like activity of Fe@Ba nanozyme using electron spin resonance (ESR) analysis with the spin-trap reagent 5-tert-butyloxycarbonyl-5-methyl-1-pyrroline N-oxide (BMPO). The distinctive quadrupling peak for BMPO-OOH confirmed the effectiveness of Fe@Ba nanozyme in scavenging superoxide anions, demonstrating the strong SOD mimetic activity, as depicted in Figure 2B. These results confirmed the effectiveness of Fe@Ba nanozyme in reducing ROS and alleviating oxidative stress. AKI is a significant side effect of DDP treatment, closely linked to the accumulation of ROS due to an overactive oxidation system and a compromised antioxidant defense. To evaluate the cellular protective effects of the Fe@Ba nanozyme, we used confocal laser scanning microscopy (CLSM) to observe the uptake of Cy5.5-labeled Fe@Ba nanozyme by DDP-induced cells over time. The increasing red fluorescence signal indicated that the nanozyme had a favorable cellular affinity and could enter mouse tubular epithelial cells (MTEC) cells to exert its therapeutic effects, as seen in Figures 2C, D; Supplementary Figure S5. A colocalization assay revealed that some Fe@Ba nanozyme could escape from lysosomes into the cytoplasm, where it scavenged ROS and exhibited antioxidant capabilities (Figure 2E). These findings indicated that Fe@Ba nanozyme can effectively be internalized by MTEC cells.

**FIGURE 3:**
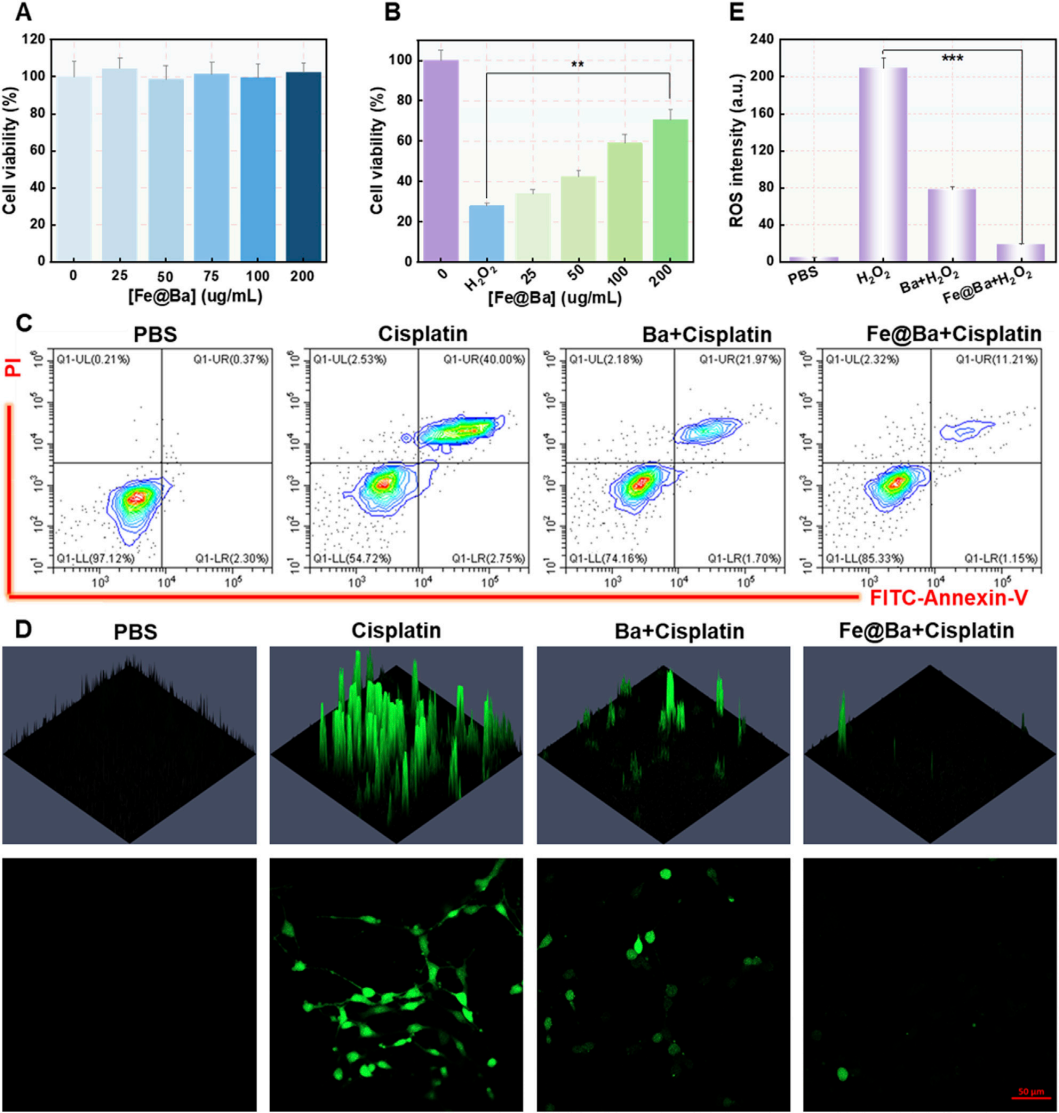
**(A)** Cell viability after a 24-h treatment with various concentrations of Fe@Ba nanozyme. **(B)** Cell viability after a 24-h treatment with various concentrations of Fe@Ba nanozyme in the presence of cisplatin. **(C)** Flow cytometry measurement of MTEC cells death rate following different treatments. **(D)** DCF fluorescence and **(E)** corresponding quantification of MTEC cells subjected to different treatments.

The protective impact of the Fe@Ba nanozyme on cells was measured using a cell counting kit 8 (CCK-8), which showed that the nanozyme did not hinder the growth of MTEC cells and was associated with strong antioxidant effects and reduction of ROS, as illustrated in Figure 3A. Importantly, the CCK-8 assay indicated that the Fe@Ba nanozyme significantly improved the MTEC cell viability in a concentration-dependent way, as shown in Figure 3B. The cytoprotective effect of the Fe@Ba nanozyme was also evaluated visually through flow cytometry analysis, with results displayed in Figure 3C; Supplementary Figure S6. These analyses showed that the Fe@Ba nanozyme-treated group had a notably higher cell survival rate compared to groups treated with free baicalein or cisplatin, aligning with the findings from the CCK-8 assay. Moreover, cellular ROS levels were quantified using a ROS probe, 2′,7′-dichlorofluorescin diacetate (DCFH-DA), as illustrated in Figures 3D, E. CLSM revealed that the Fe@Ba nanozyme had a greater capacity to reduce ROS than baicalein alone, attributed to its robust catalytic activities and antioxidant properties. These results confirm that the Fe@Ba nanozyme can effectively lower cellular ROS levels and enhance cell viability.

**FIGURE 4:**
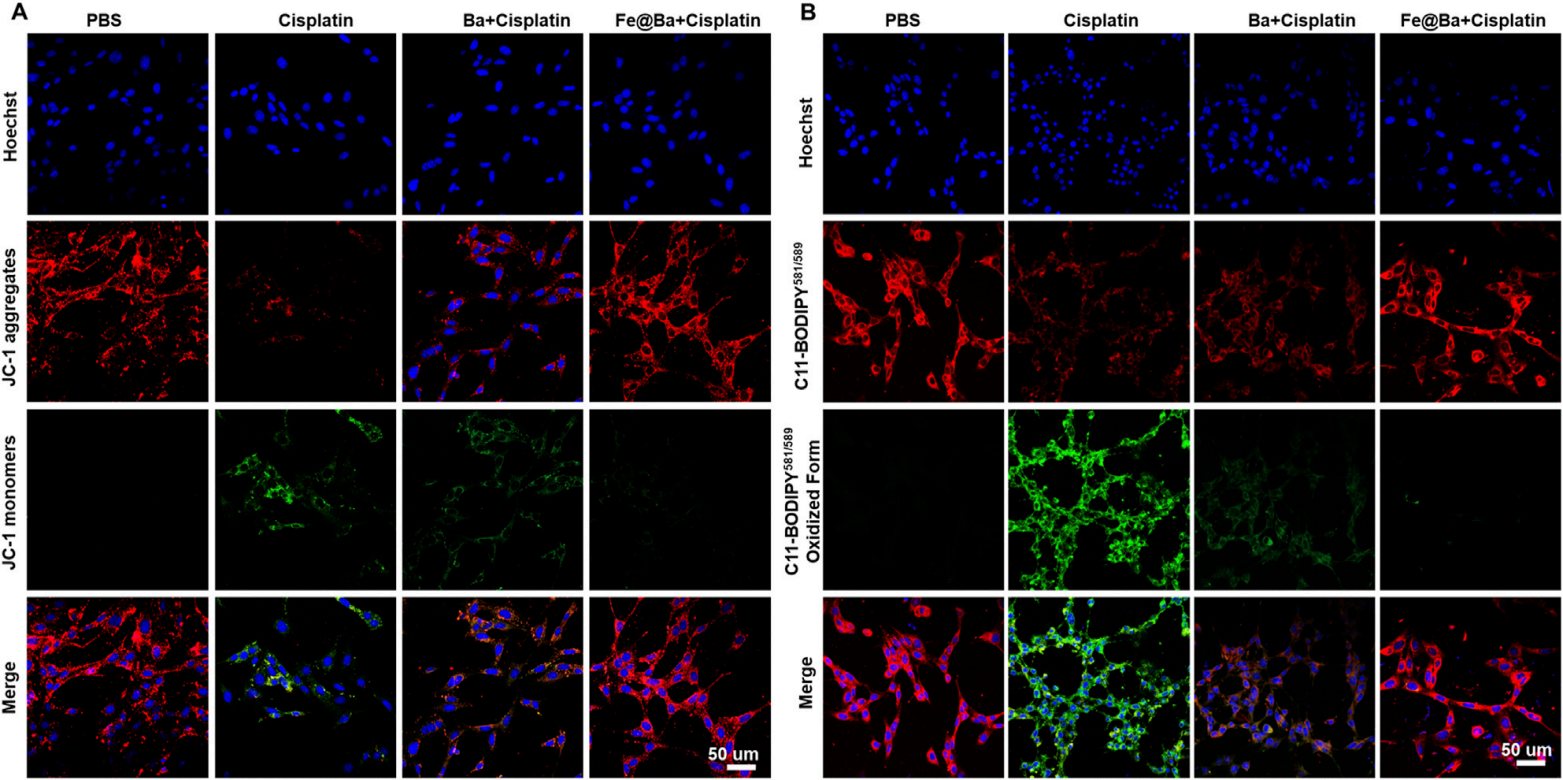
**(A)** Confocal images of MTEC cells stained with JC-1 kit following a 24-h treatment with different formulations. **(B)** Confocal images of MTEC cells stained with C11-BODIPY^581/589^ following different formulations.

**FIGURE 5:**
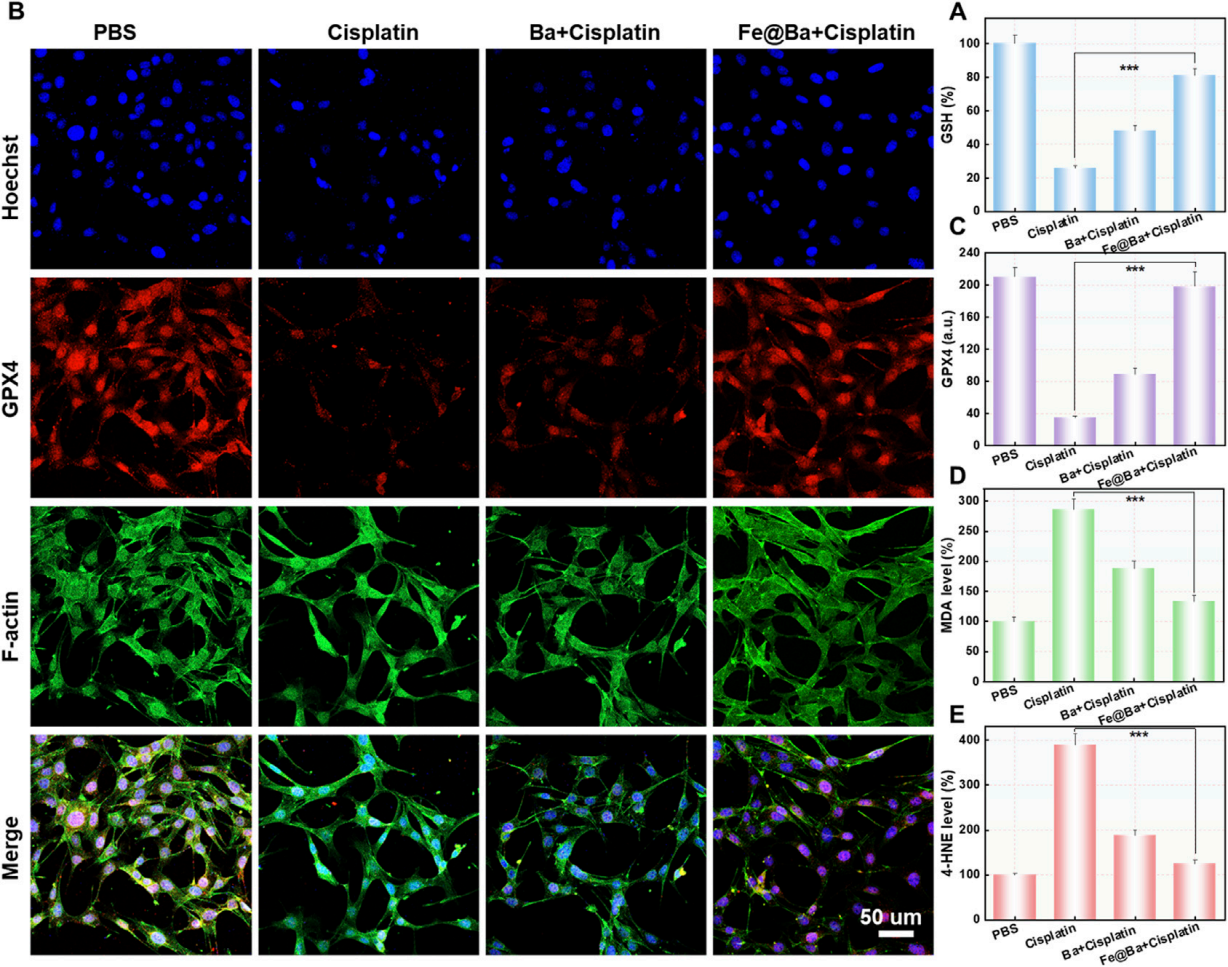
**(A)** The cellular levels of GSH after different treatments were measured using a DTNB assay kit. **(B)** Confocal images **(C)** and the corresponding quantification (f) of GPX4 expression in MTEC cells after different formulations. **(D)** Quantitative analysis of MDA **(E)** and 4-HNE levels following different formulations.

To understand how the Fe@Ba nanozyme can prevent ferroptosis in MTEC cells, we used a fluorescence probe, 5,5′,6,6′-tetrachloro-1,1′,3,3′-tetraethyl-imidacarbocyanine iodide (JC-1), to assess changes in mitochondrial membrane potential (MMP). The fluorescence of probe shifts from green to red as MMP decreases. CLSM images revealed a decrease in red fluorescence and an increase in green fluorescence in the DDP group, indicating MMP damage, as shown in Figure 4A. In contrast, the Fe@Ba nanozyme treatment resulted in a decrease in green fluorescence and an increase in red fluorescence, suggesting significant mitochondrial depolarization. Ferroptosis is marked by mitochondrial damage, and we used the fluorescent probe BODIPY C11^581/591^ to measure lipid peroxide levels, which change from red to green fluorescence. CLSM images indicated that the Fe@Ba nanozyme significantly reduced green fluorescence and increased red fluorescence, as depicted in Figure 4B, suggesting a reduction in lipid peroxides and thus inhibiting LPO. Furthermore, the Fe@Ba nanozyme was found to enhance glutathione (GSH) levels, which in turn increases the expression of GPX4, a key enzyme that inhibits ferroptosis. Figure 5A shows that the Fe@Ba nanozyme substantially increased GSH levels due to its excellent enzymatic activities and antioxidant capabilities. An immunofluorescence assay was used to measure GPX4 expression, and Figures 5B, C shows that the Fe@Ba nanozyme upregulated GPX4 expression, likely due to its catalytic activities in raising GSH levels. We also measured additional indicators of ferroptosis, malondialdehyde (MDA) and 4-hydroxynonenal (4-HNE), to confirm the inhibitory effect of the Fe@Ba nanozyme on ferroptosis. Figures 5D, E demonstrate that MTEC cells treated with the Fe@Ba nanozyme significantly reduced the levels of these harmful byproducts. These findings suggest that the Fe@Ba nanozyme can effectively neutralize ROS and protect mitochondria from depolarization, potentially offering antioxidant protection in an AKI model. This provides strong evidence for the potential of Fe@Ba nanozyme as an effective inhibitor of ferroptosis.

**FIGURE 6:**
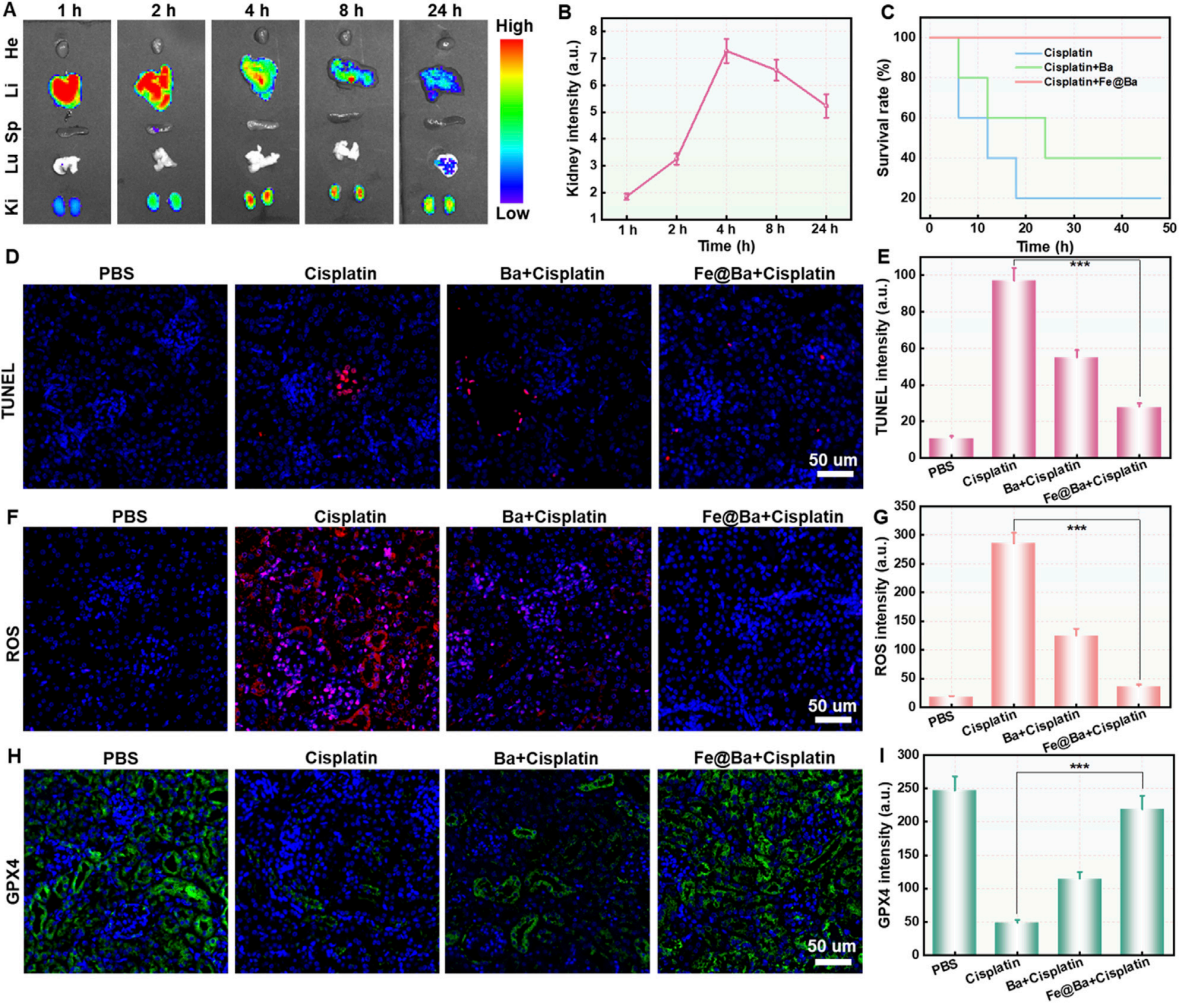
**(A)** Fluorescence images **(B)** corresponding quantification of cisplatin-induced AKI mice at different time points post-injection with Cy5.5-labeled Fe@Ba nanozyme. **(C)** Kaplan-Meier survival curves of mice following different treatments. **(D)** TUNEL staining of kidney slices and **(E)** the corresponding quantification from various groups following a 24-h treatment with different formulations. **(F)** ROS staining and **(G)** the corresponding quantification of kidney slices following various formulations. **(H)** Immunofluorescence staining and **(I)** the corresponding quantification of GPX4 in kidney slices following various formulations.

The animal study protocol was approved by the Ethical Committee of Fujian Medical University (IACUC FJMU 2024-Y-0291). We used an IVIS imaging system to track the *in vivo* distribution of the Cy5.5-labeled Fe@Ba nanozyme. The imaging showed that the fluorescence signal at the kidney area increased over time, peaking 24 h after injection, which corresponds to ROS scavenging and ferroptosis inhibition effects of Fe@Ba nanozyme, as seen in Figures 6A, B. The mice were divided into four groups: PBS control, DDP treatment, DDP plus free Ba, and DDP plus Fe@Ba nanozyme. The results, as depicted in Figure 6C, indicated that the Fe@Ba nanozyme group had a significantly extended survival rate compared to the free Ba group, demonstrating the nanozyme’s superior antioxidant performance. We have measured the serum creatinine and blood urea nitrogen of different treatment groups in mice *in vivo*. As shown in Supplementary Figures SS7, S8, Fe@Ba nanozyme can significantly decrease the levels of serum creatinine, blood urea nitrogen, and inflammatory factor. To further confirm the antioxidant effect of Fe@Ba nanozyme, we performed terminal deoxynucleotidyl transferase-mediated dUTP nick-end labeling (TUNEL) staining on kidney sections. Figures 6D, E; Supplementary Figure S9 show that kidneys treated with the Fe@Ba nanozyme experienced less damage than those treated with free Ba, indicating a significant therapeutic benefit. Immunofluorescence staining confirmed the inhibition of kidney ferroptosis, with notable reductions in ROS and increases in GPX4 expression observed directly in the kidney, as shown in Figures 6F–I and S10-S11. In conclusion, the Fe@Ba nanozyme reduced lipid peroxidation, decreased intracellular oxidative stress, and enhanced GPX4-mediated protection against ferroptosis. Blood biochemistry analysis of mice and H&E staining further confirmed the antioxidant ability of Fe@Ba nanozyme, significantly reducing the levels of blood urea nitrogen (BUN), creatinine (CREA), and renal injury (Supplementary Figures S12, S13). These findings underscore the potential of Fe@Ba nanozyme as an effective inhibitor of ferroptosis for the treatment of AKI.

## Conclusion

We successfully developed a metal-polyphenol self-assembling nanozyme as a ROS scavenger for treating cisplatin-induced AKI. The Fe@Ba nanozyme was synthesized through the interaction between Fe^3+^ and the antioxidant compound Ba, derived from traditional Chinese medicine. This nanozyme effectively inhibited LPO, a key driver of ferroptosis, by scavenging highly reactive ROS and enhancing antioxidant properties of Ba. Fe@Ba nanozyme exhibited CAT- and SOD-like activities to convert toxic ·O_2_^−^ into harmless oxygen, thereby reducing inflammation and preventing ferroptosis. Additionally, Fe@Ba nanozyme upregulated the expression of GPX4, further inhibiting ferroptosis. Fluorescence imaging demonstrated that Cy5.5-labeled Fe@Ba nanozyme effectively accumulated in the kidneys. *In vivo* experiments confirmed that Fe@Ba nanozyme reduced inflammation and improved survival rates in an AKI model, showcasing its therapeutic potential. This research not only validated the concept of self-assembling nanozymes but also offered new insights into the use of nanomedicine for AKI treatment, emphasizing their ability to counteract inflammation-related damage.

## Data Availability

The original contributions presented in the study are included in the article/Supplementary Material, further inquiries can be directed to the corresponding authors.
