# Supplementary material for: Metal-phenolic nanozyme as a ferroptosis inhibitor for alleviating cisplatin-induced acute kidney injury
Source: Front Pharmacol. 2025 Apr 1;16:1535969. doi: 10.3389/fphar.2025.1535969 (PMC11996903; doi:10.3389/fphar.2025.1535969)
Supplement: Supplementary file 1 [file DataSheet1.pdf]

Supporting Information

Metal-phenolic Nanozyme as A Ferroptosis Inhibitor for Alleviating Cisplatin-induced Acute Kidney Injury

Yunfeng Xiong[a†], Huimin Kang[b†], Yanping Rao[a], Xiayu Huang[a], Yang Zhu[c*], Lixin Wei[a,d*]

[a] Department of Nephrology, Fujian Medical University Union Hospital, Fuzhou, 350001, China.

[b] Department of Pediatrics, Fujian Medical University Union Hospital, Fuzhou, 350001, China.

[c] CAS Key Laboratory of Soft Matter Chemistry, Department of Chemistry, University of Science and Technology of China, Hefei 230026

[d] Fujian Institute of Clinical Immunology, Fuzhou, 350001, China.

[†]These authors contributed equally to this work.

Materials

Ferric chloride ($FeCl_3$), Baicalein (Ba), polyvinylpyrrolidone (PVP) paraformaldehyde (PFA), Tween 20, Triton X-100 and hydrogen peroxide (H_2O_2) were purchased from Sinopharm Chemical Reagents (Shanghai, China). Methylene blue (MB), 3,3',5,5'-tetramethylbenzidine (TMB), 5,5′-dithiobis (2-nitrobenzoic acid) (DTNB), and C11-BODIPY$^{581/591}$ were provided by Sigma-Aldrich (St. Louis, USA). Hoechst, 2′,7′-dichlorofluorescin diacetate (DCFH-DA), annexin V-FITC/PI apoptosis detection kit, cell counting kit 8 (CCK-8), and 1,1',3,3'-tetraethyl-5,5',6,6'-tetrachloroimidacarbocyanine iodide (JC-1) were bought from Beyotime (Shanghai, China). Dulbecco's modified eagle medium (DMEM) was purchased from Hyclone (Logan, USA). 5,5-dimethyl-1-pyrroline N-oxide (DMPO) was bought from Dojindo (Dojindo, China). Live & Dead Bacterial Staining Kit (Cat#40274ES60) and GMyc-PCR Mycoplasma Test Kit (Cat#40601) was purchased from Yeasen Biotechnology (Shanghai) Co., Ltd.. 20 mm glass-bottom dishes, and centrifuge tubes were obtained from NEST Biotechnology Co. Ltd. (Wuxi, China). Deionized (DI) water was obtained from a Milli-Q water purification system.

Methods

Synthesis of Fe@Ba nanozyme

The Fe@Ba nanozyme was constructed via a self-assembled strategy-driven nanoprecipitation. 5 mg $FeCl_3$ and 50 mg PVP were dissolved 20 mL DI H_2O, ultrasound for 10 min. Then 5 mg Ba dissolved in DMSO was dropwise added above solution under stirring. After 2 h, the product was collected by centrifugation and washed with DI H_2O for 3 times.

SOD-like activity

The SOD-like catalytic activity of Fe@Ba nanozyme was assessed using a colorimetric assay. 100 ug/mL Fe@Ba nanozyme were mixed with 200 μL of WST working solution. Then 20 μL of enzyme working solution was added to initiate the reaction. The plate was incubated at 37°C for 30 min. Absorbance was measured at 450 nm using a microplate reader

CAT-like activity

The catalase-like activity of Fe@Ba nanozyme was evaluated using a dissolved oxygen analyzer. 50 mM H_2O_2 was dissolved in buffer solution, and 100 ug/mL Fe@Ba nanozyme was added to the solution. Oxygen concentration was immediately and continuously measured for 5 min.

Cellular uptake

MTEC cells were seeded into confocal dishes and incubated overnight. The medium was then replaced with 100 μg/mL Fe@Ba nanozyme, and cells were incubated for different time points (0, 1, 2, and 4 h). After incubation, the cells were treated with DMEM containing 20 ug/mL Hoechst and 10 uM Lyso-tracker for 30 min. CLSM was performed to image.

In Vitro therapeutic effect of Fe@Ba nanozyme

Cell-viability was determined by the CCK-8 assay and flow cytometry analysis. For CCK-8 assay, MTEC cells were planted for 24 h. Then, the cells were incubated with various concentrations of Fe@Ba nanozyme or Fe@Ba nanozyme with cisplatin. After treatment for 24 h, the medium was replaced with fresh medium containing 10 μL CCK-8 and quantified by the absorbance at 450 nm using a microplate reader.

Flow cytometry analysis

MTEC cells were seeded in 6-well plates and incubated overnight. The DMEM containing 100 ug/mL Fe@Ba nanozyme was added. After 12 h of incubation, the cells were stained using Annexin-V and PI according to the manufacturer's protocol and measured using a flow cytometry.

ROS scavenged by Fe@Ba nanozyme

MTEC cells were seeded in confocal dish. After incubation for 12 h, the cells were treated with various formulation for 4 h. Then, the cells were co-stained with DCFH-DA (10 μM) and Hoechst (10 μM). After 20 minutes of incubation, the fluorescence imaging of cells was imaged by confocal microscopy.

Measurement of mitochondrial membrane potential

To investigate the MMP, MTEC cells were seeded and incubated for 24 h. Subsequently, the cells were exposed to Fe@Ba nanozyme. Then the cells were treated according to the JC-1 kit. The fluorescence imaging of cells was analyzed by CLSM.

Lipid peroxidation

MTEC cells were seeded and incubated for 24 h. Subsequently, the cells were exposed to Fe@Ba nanozyme. Then the cells were stained with BODIPY$^{581/591}$-C11 probe and Hoechst for 30 min. The fluorescence imaging of tumor cells was imaged by confocal.

In vivo therapeutic effect of Fe@Ba nanozyme

Animal experiments were performed according to the protocol approved by The Ethical Committee of Fujian Medical University. The balb/c mice were randomly divided into four groups (5 mice per group) and intraperitoneal administrated with 20 mg/kg cisplatin to construct AKI model. After 12 h of treatment, the mice were intravenously administrated with 10 mg/kg Ba or Fe@Ba nanozyme. After 3 days of different

treatments, the mice were euthanatized for histological examination.

Statistical analysis

All quantitative data were expressed as the mean ± standard deviation (SD). Statistical analyses were performed using the Student's two-tailed t-test (*$P < 0.05$, **$P < 0.01$, ***$P < 0.001$).

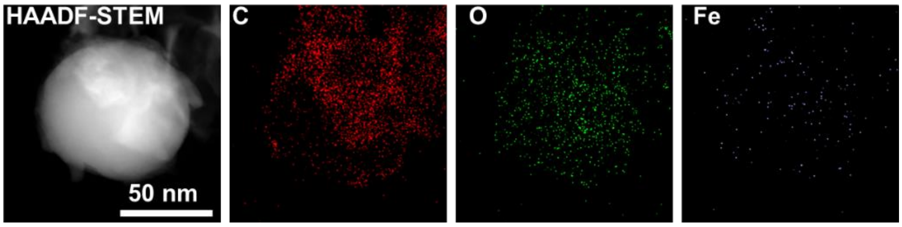

Figure S1. The HAADF-STEM image of Fe@Ba nanozyme.

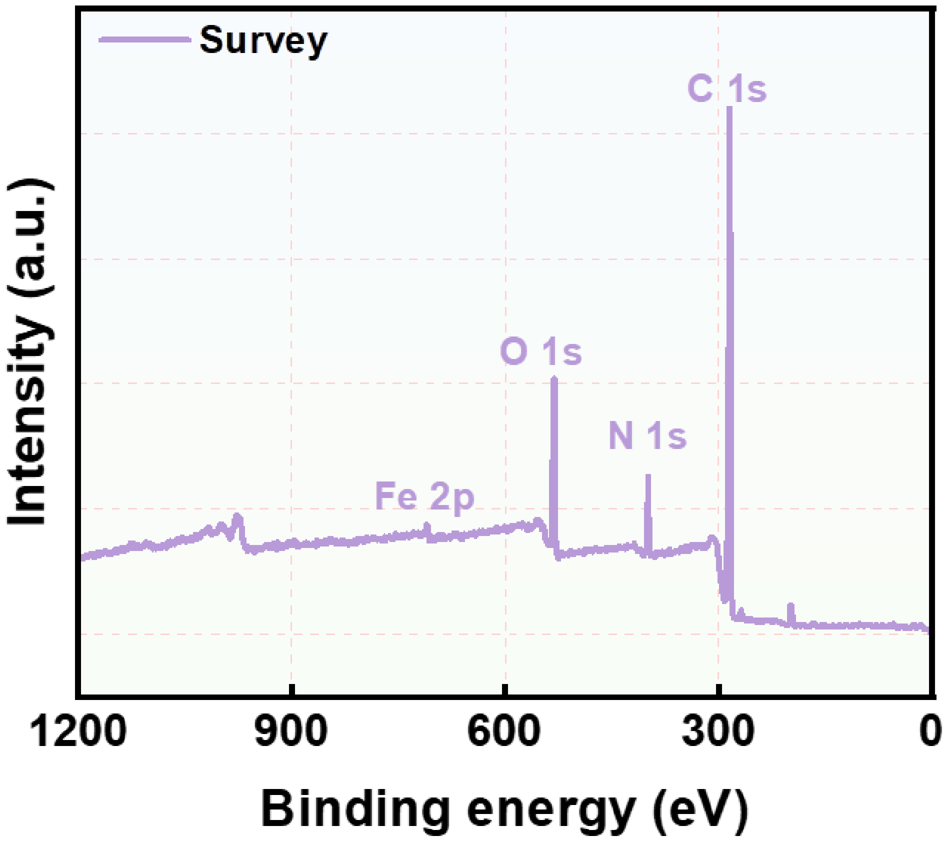

Figure S2. The survey XPS spectrum of Fe@Ba nanozyme.

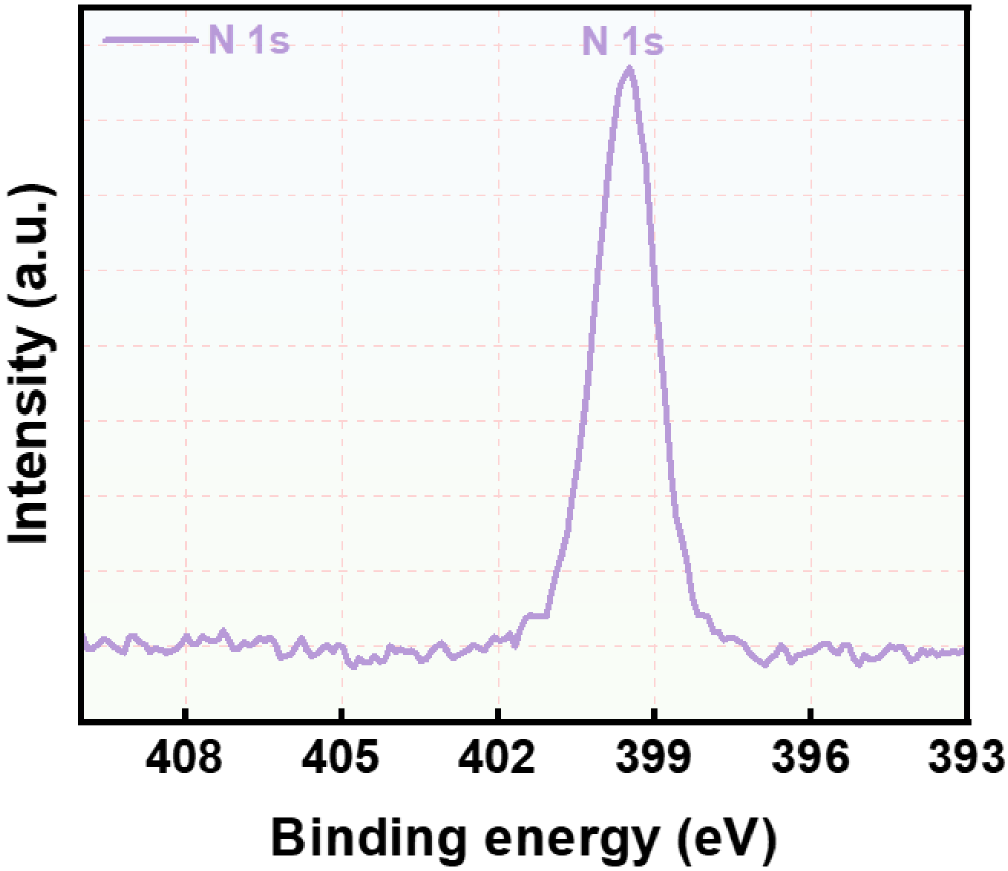

Figure S3. The N 1s XPS spectrum of Fe@Ba nanozyme.

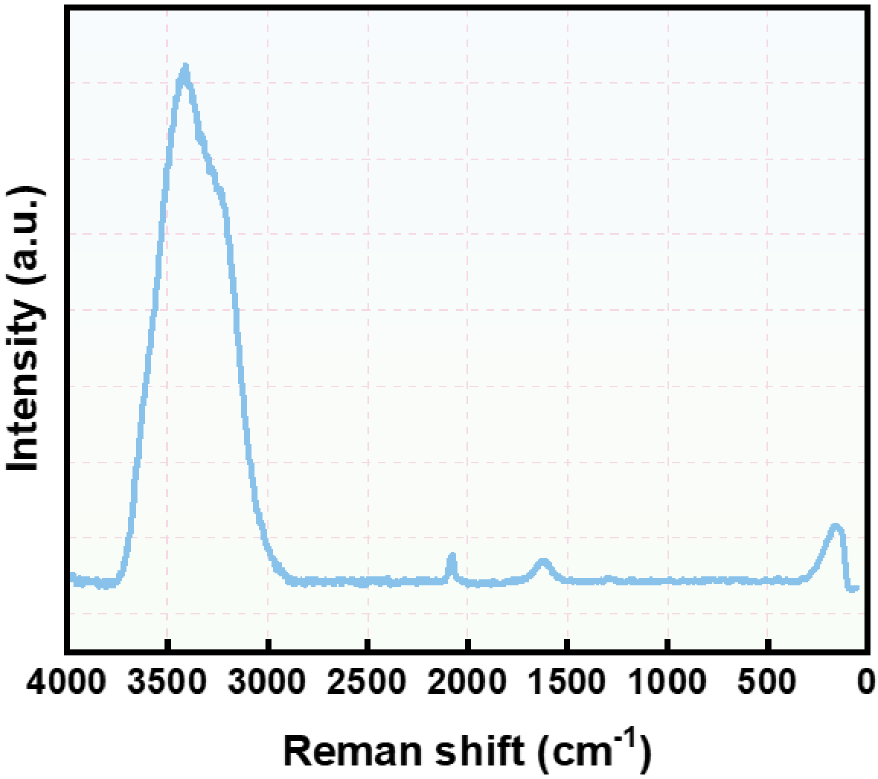

Figure S4. The Raman spectrum of Fe@Ba nanozyme.

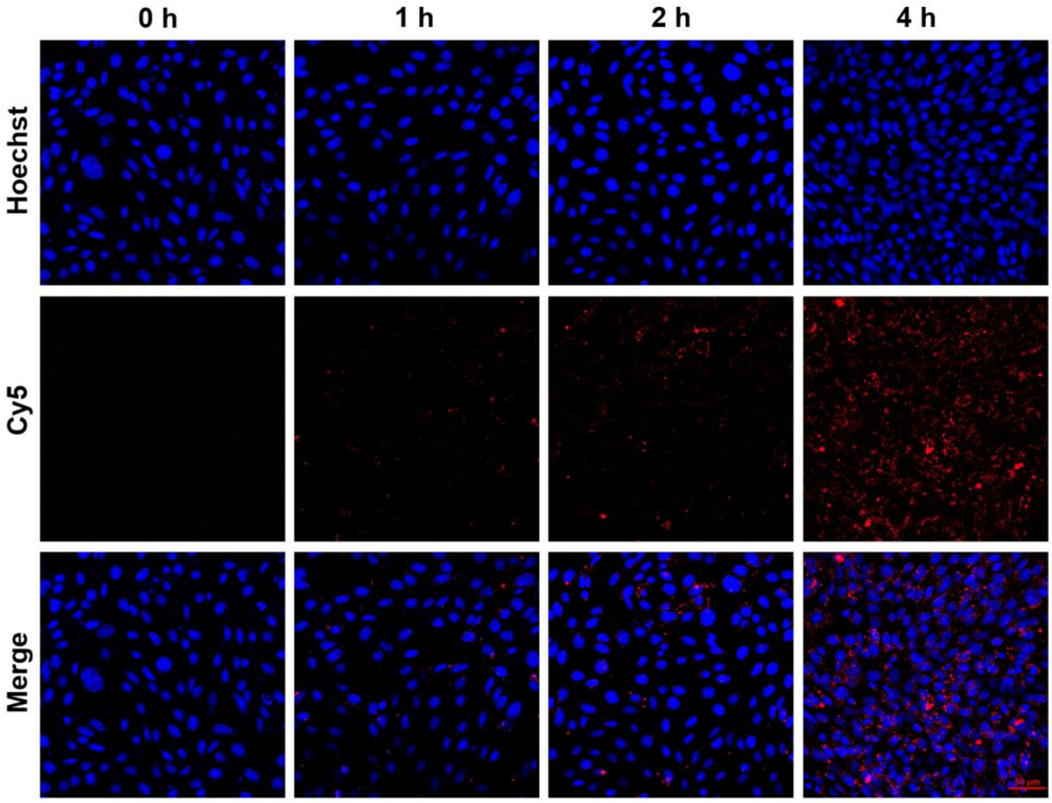

Figure S5. The CLSM images of MTEC cells treated with Cy5.5-labeled Fe@Ba nanozyme.

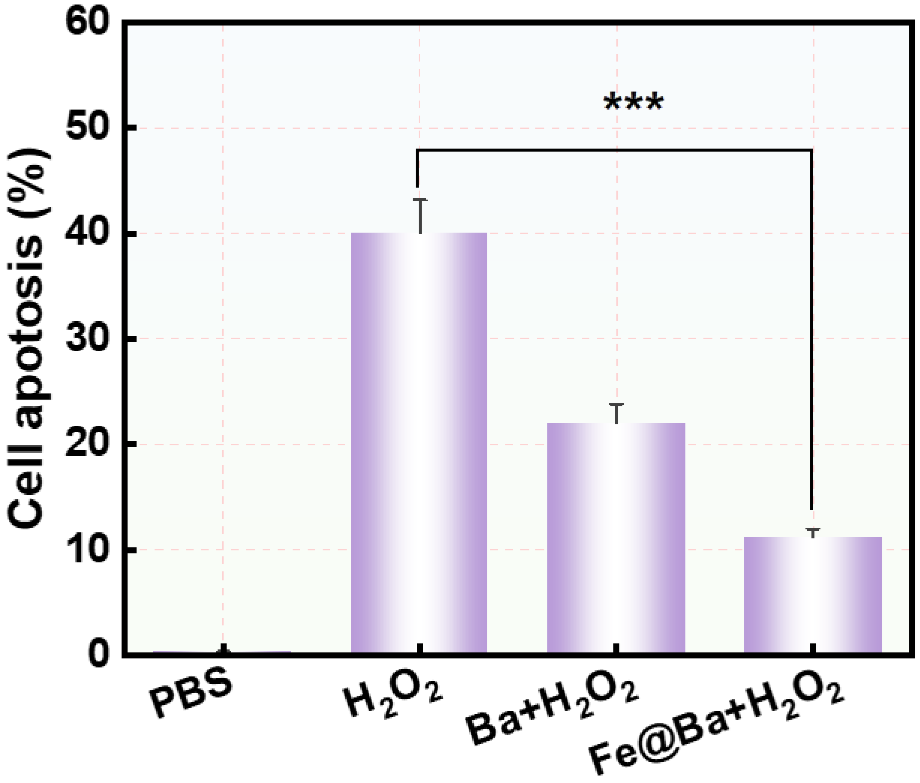

Figure S6. Flow cytometry measurement of MTEC cells death rate following different treatments.

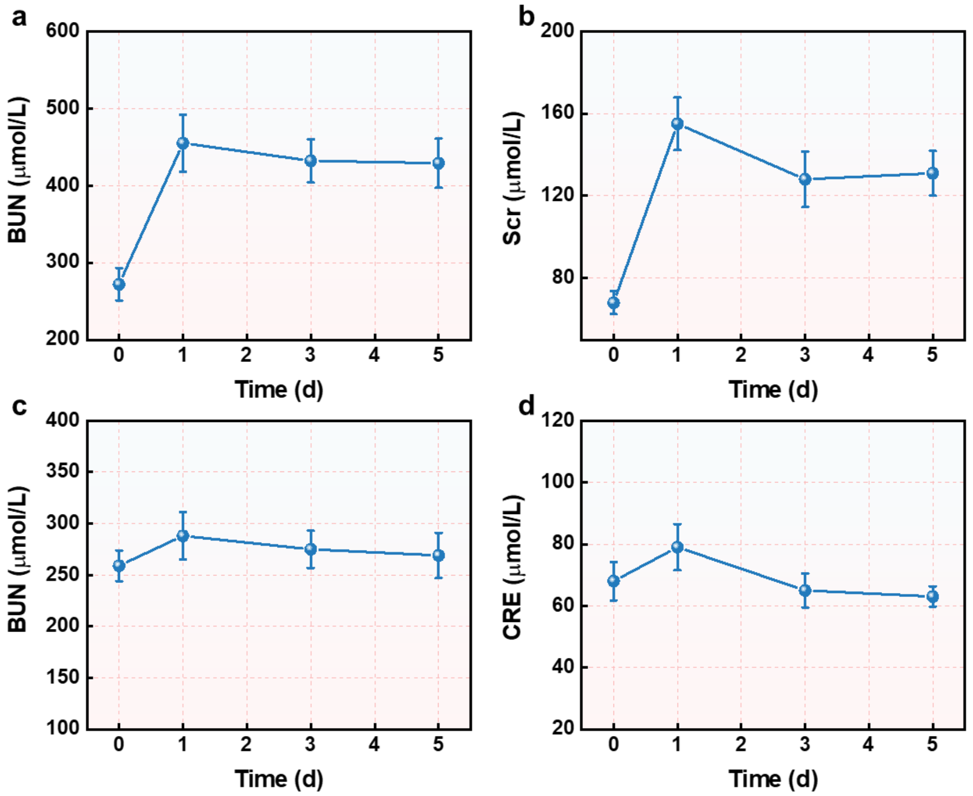

Figure S7. The serum creatinine and blood urea nitrogen of different treatment groups in mice in vivo. (a) and (b) for Cisplatin group, (c) and (d) for Cisplatin plus Fe@Ba nanozyme group.

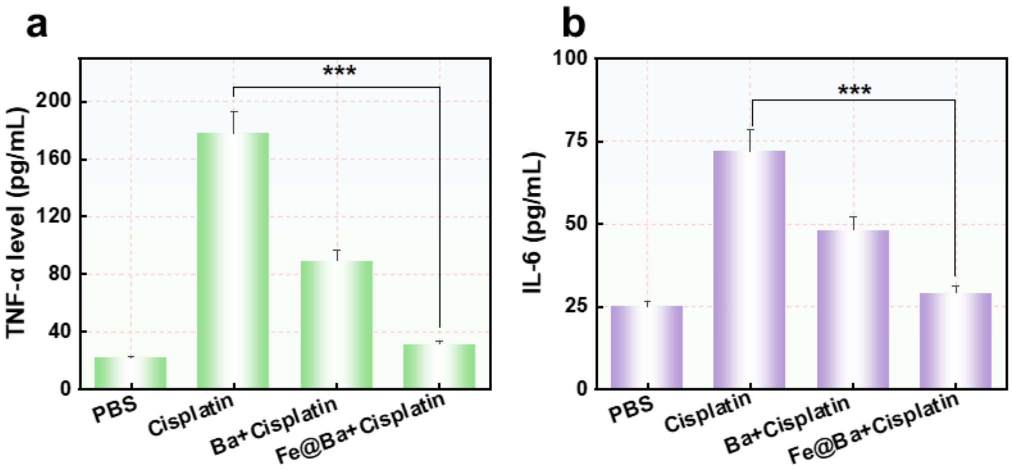

Figure S8. The TNF-α and IL-6 levels in different groups measured by ELISA.

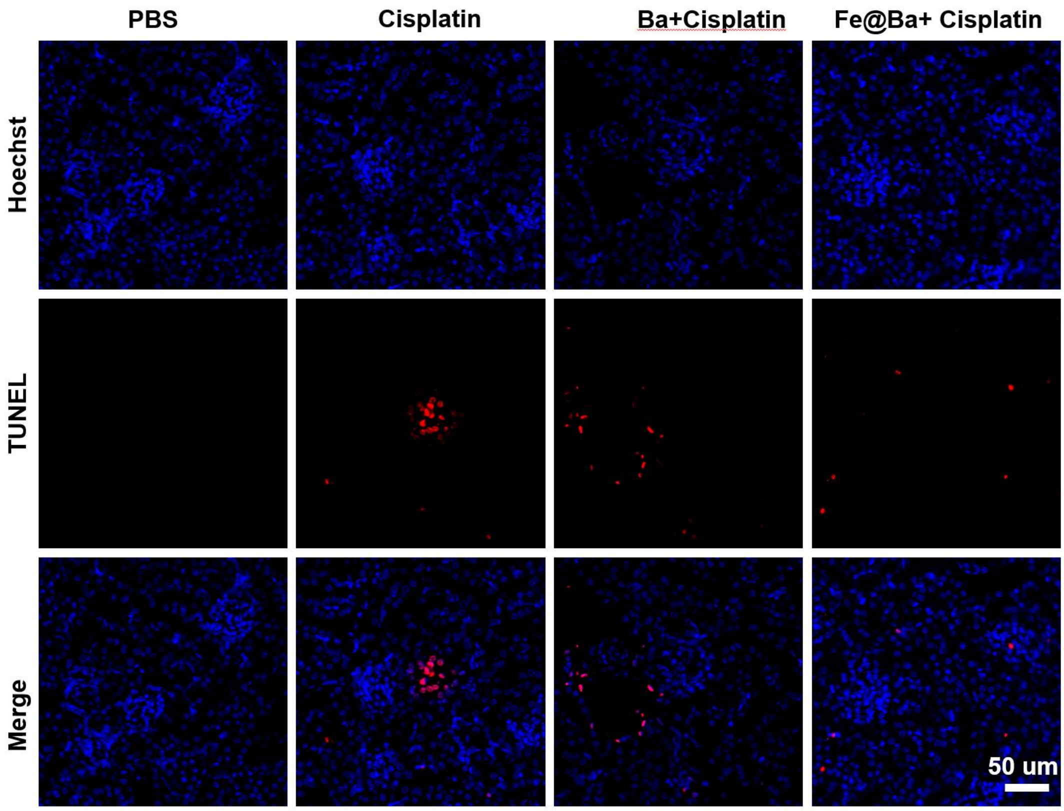

Figure S9. TUNEL staining of tumor slices from various groups following a 24-hour treatment with different formulations.

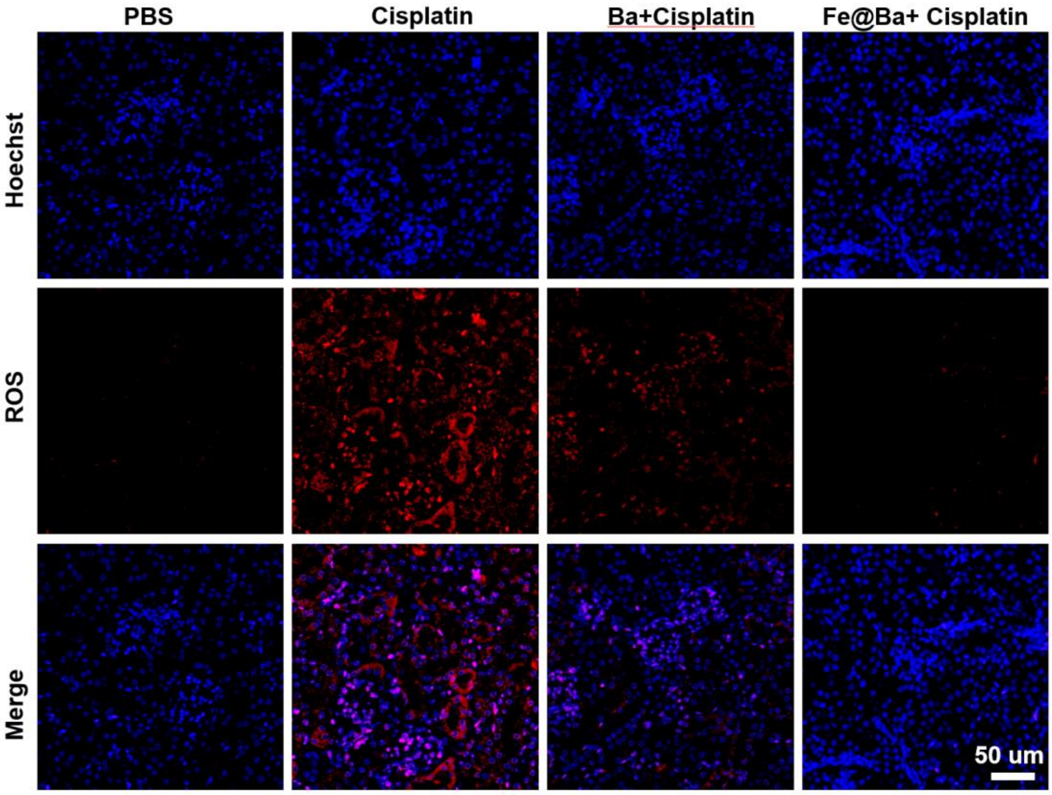

Figure S10. ROS staining of kidney slices following various formulations.

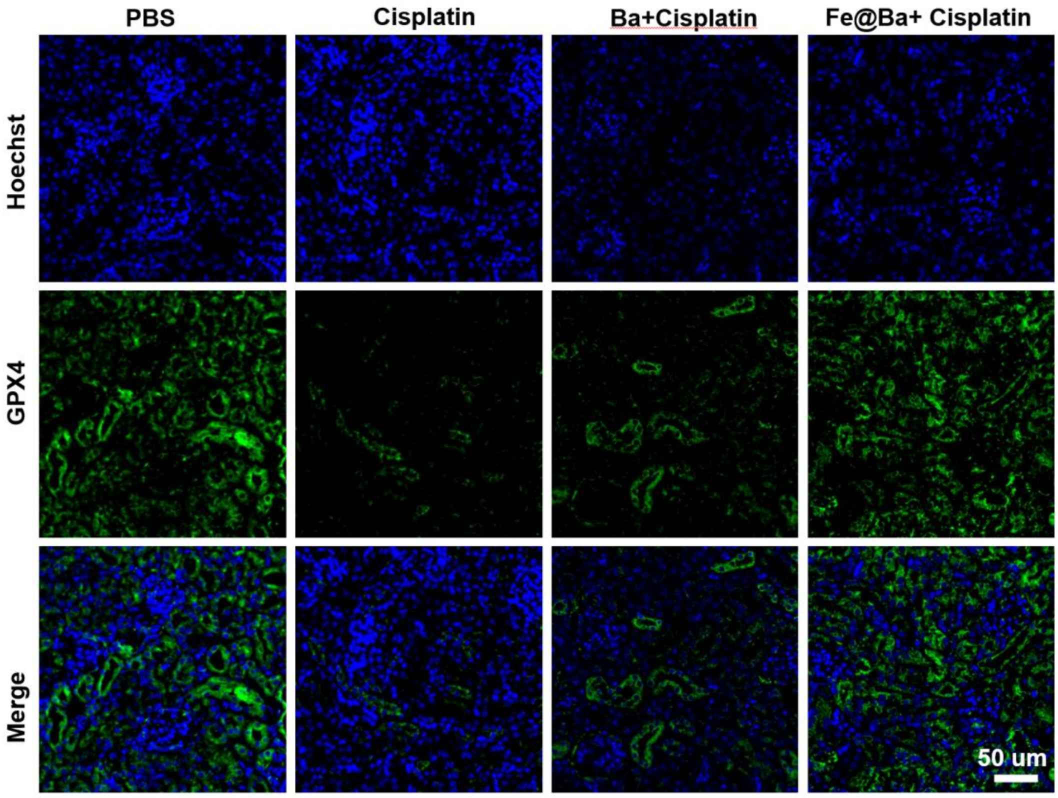

Figure S11. Immunofluorescence staining of GPX4 in kidney slices following various formulations.

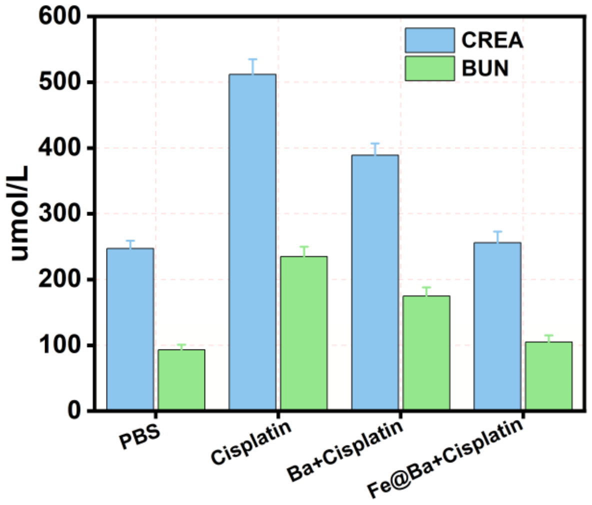

Figure S12. Blood biochemistry analysis of mice after intravenous injection with different formulations. Blood urea nitrogen (BUN) and Creatinine (CREA).

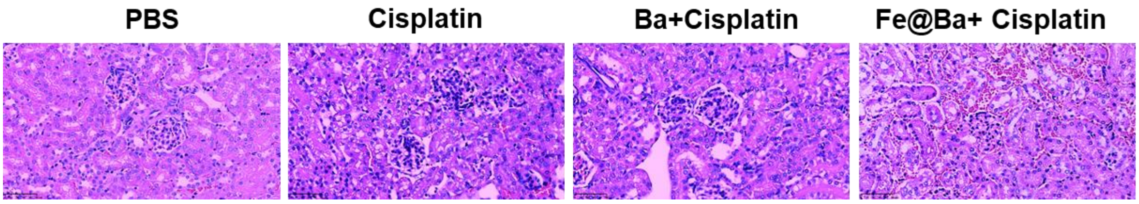

Figure S13. H&E staining of mice after intravenous injection with different formulations.
